# Supplementary material for: Interplay of structural chirality, electron spin and topological orbital in chiral molecular spin valves
Source: Nat Commun. 2023 Aug 24;14:5163. doi: 10.1038/s41467-023-40884-9 (PMC10449876; doi:10.1038/s41467-023-40884-9)
Supplement: Supplementary file 1 — Supplementary Information [file 41467_2023_40884_MOESM1_ESM.docx]

**SUPPLEMENTARY INFORMATION**

**Interplay of Structural Chirality, Electron Spin and Topological Orbital in Chiral Molecular Spin Valves**

Yuwaraj Adhikari^1,+^, Tianhan Liu^1,+^, Hailong Wang^2^, Zhenqi Hua^1^, Haoyang Liu^1^, Eric Lochner^1^, Pedro Schlottmann^1^, Binghai Yan^3,*^, Jianhua Zhao^2,*^, Peng Xiong^1,*^

^1^ Department of Physics, Florida State University, Tallahassee, Florida 32306, USA

^2^ State Key Laboratory of Superlattices and Microstructures, Institute of Semiconductors, Chinese Academy of Sciences, Beijing 100083, China

^3^ Department of Condensed Matter Physics, Weizmann Institute of Science, Rehovot, Israel

^+^ These authors contributed equally.

^*^ emails: [binghai.yan@weizmann.ac.il](mailto:binghai.yan@weizmann.ac.il), [jhzhao@semi.ac.cn](mailto:jhzhao@semi.ac.cn), [pxiong@fsu.edu](mailto:pxiong@fsu.edu)

1. I-V of molecular junctions with Au and Al electrode


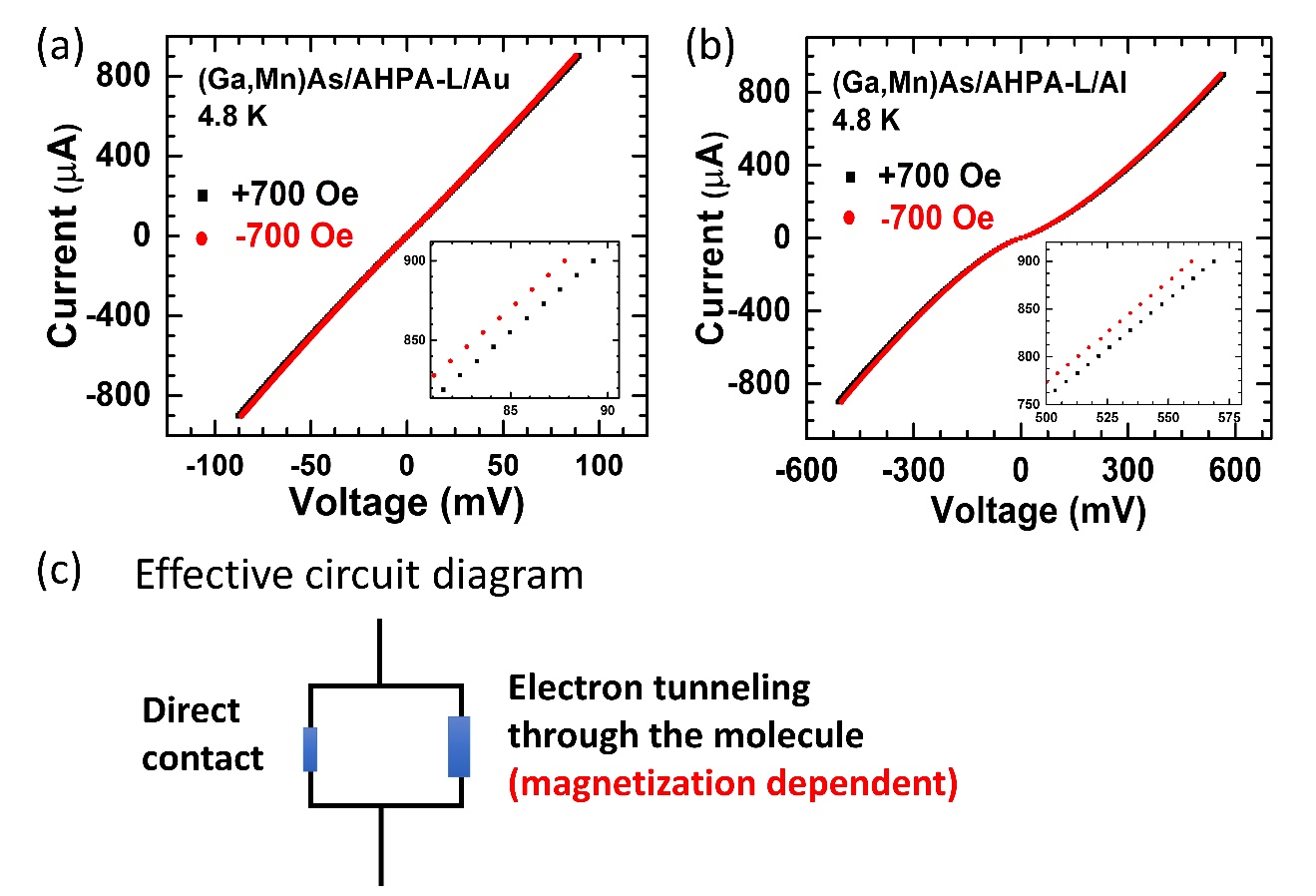


**Supplementary Fig. 1**: The I-V curves of the chiral molecular (AHPA-L) junctions with **(a)** Au and **(b)** Al contact in perpendicular magnetic fields of ±700 Oe. **(c)** The effective circuit diagram of a molecular junction showing parallel conduction of current through the direct contact of normal metal (NM) electrode and (Ga,Mn)As and through chiral molecules. Insets of (a) and (b) show the close-up images of the respective I-V’s.

The field dependent I-V curves for two chiral molecular junctions with AHPA-L SAM and Au and Al contacts are shown in Supplementary Fig. 1(a) and 1(b), respectively. The split between the I-V curves in perpendicular saturation fields of opposite polarities, ±700 Oe, is shown more clearly in the close-up images for the Au and Al junctions, respectively.

We note some apparent differences between the I-V curves for the Au junction here and a similar device in our previous work [1]: Here the I-V’s are much more linear and the bifurcation is much less obvious. We believe the differences stem from a larger contribution to the current from the parallel contribution through direct contact between the Au and (Ga,Mn)As, as illustrated in Supplementary Fig. 1(c).

1. Spin valve conductance (ΔG) versus total junction conductance (G)


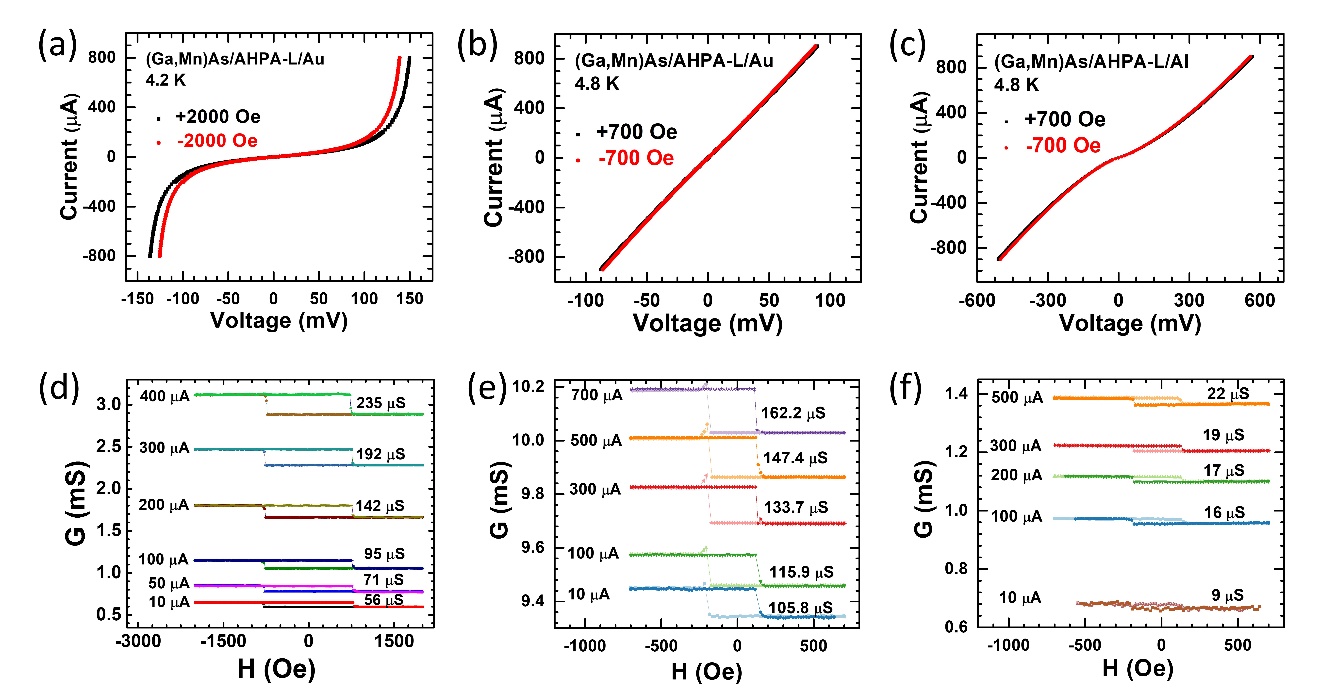


**Supplementary Fig. 2:** **(a), (b), (c)** I-V curves for a (Ga,Mn)As/AHPA-L/Au junction from our previous work [1], and a (Ga,Mn)As/AHPA-L/Au junction and a (Ga,Mn)As/AHPA-L/Al junction in the present work, respectively. **(d) - (e)** show the corresponding MC measurements for the three junctions. (a) and (e) is reprinted with the permission of [1]. Copyright (2020) American Chemical Society.

As was elaborated previously [1] and emphasized again in the main text here, because of the contribution to the total current from the parallel conduction [Supplementary Fig. 1(c)], the I-V and **total** conductance of the molecular junctions of similar structures may vary greatly depending on the degree of parallel conduction (quality of the SAM assembly), however, both the magnitude and bias current dependence of the CISS spin valve conductance (ΔG) of the junctions are found to be consistently similar. This is well illustrated in Supplementary Fig. 2, which shows the I-V and magnetoconductance measurements for a (Ga,Mn)As/AHPA-L/Au junction in our previous work  [1], and the (Ga,Mn)As/AHPA-L/Au(Al) junctions in this work. *Clearly, the zero-bias total junction conductance of the Au junction in the previous work is much* ***smaller*** *than that of the Au junction in this work, and even smaller than that of the Al junction here, whereas the ΔG for that junction are* ***comparable*** *to those of the Au junction in the present work at low bias currents (e.g., 100 μA), and becomes significantly* ***greater*** *at high biases.* We believe the more nonlinear I-V’s and smaller total junction conductance in the Au junction in the previous work are signatures of a smaller contribution to the total current from the parallel contribution through direct contact between the Au and (Ga,Mn)As. This conclusion is also corroborated by the more rapid increase of ΔG with the bias current seen in the first Au junction.

In summary, these observations are compelling evidence that *the total G is spurious and has no bearing on CISS spin-valve conductance;* *it is the ΔG that truly reflects the CISS effect*. The fact that different Au junctions show large variations of the I-V and total G, but exhibit similar bias-dependent ΔG, lends further credence to our model and the associated analyses and conclusion.

1. **Other normal metal (NM) electrode materials of varying SOC strengths**


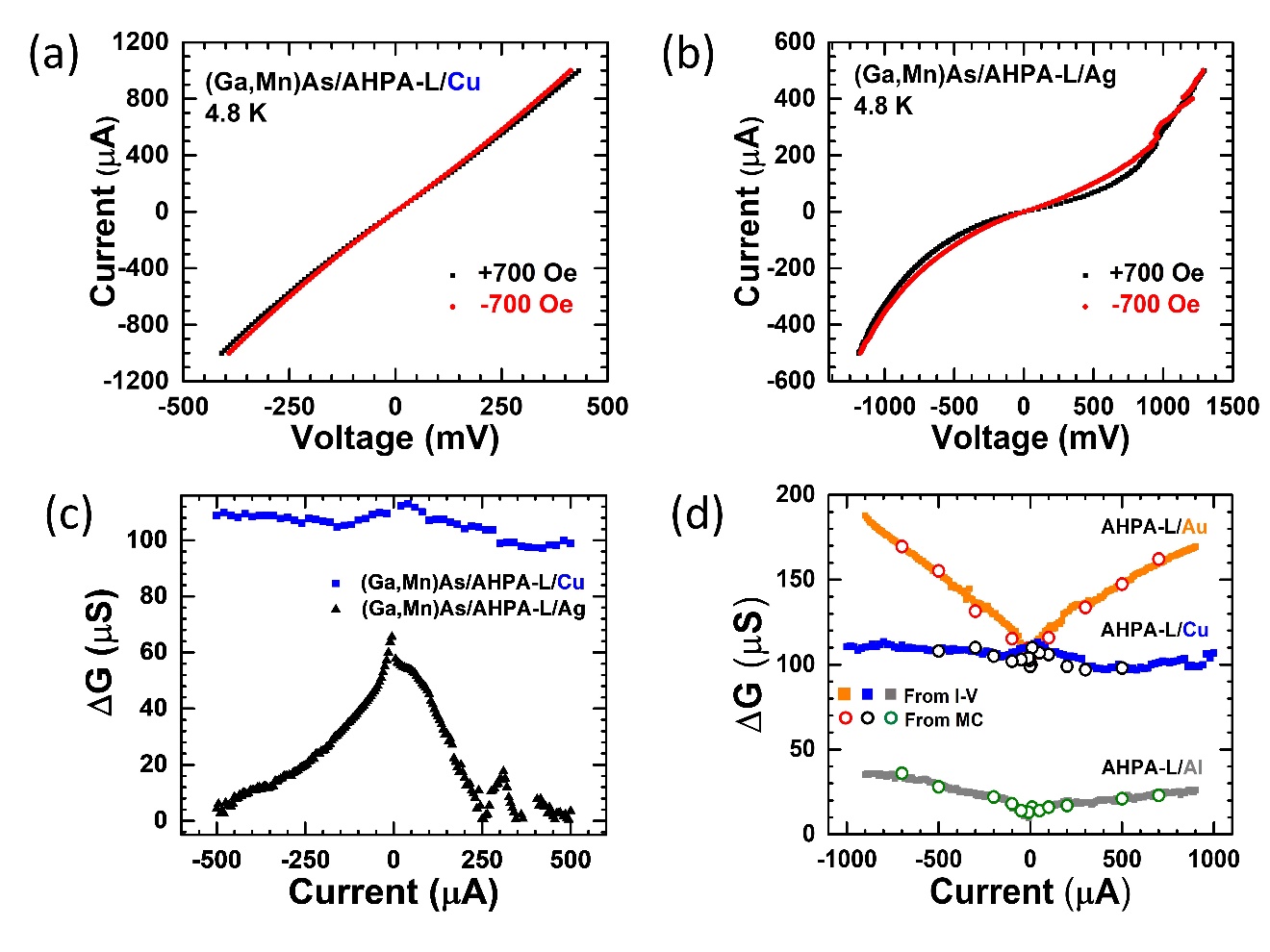


**Supplementary Fig 3:** The I-V curves for chiral molecular (AHPA-L) junctions with **(a)** Cu, and **(b)** Ag, contact in perpendicular magnetic fields of ±700 Oe. **(c)** The bias dependence of ΔG for the junctions with Cu and Ag contact extracted from the I-V curves. **(d)** Comparison of the bias-current dependent ΔG for the junctions with Au, Cu. and Al contact. The solid squares are ΔG extracted from I-V curves, and the open circles are ΔG from MC measurements at different bias currents.

We have fabricated and measured large numbers (>10) of devices with Cu and Ag electrodes, two NM materials of intermediate SOC strengths between Au and Al. However, despite the repeated attempts, for either material, we were unable to obtain results with the degree of consistency seen in Au and Al devices. We speculate that this originated from poor interfaces of the molecular SAM with Cu and Ag, possibly due to the higher evaporation temperatures or some specific chemistry with the organic molecules for Cu and Ag. Supplementary Fig. 3 shows a representative I-V and resulting ΔG from a Ag junction, and a **best** set from the Cu junctions. Although the magnitude of ΔG for this Cu junction falls well between those of the Au and Al junctions, as shown in Supplementary Fig. 3(d), it does not exhibit the bias-current dependence consistently seen in Au and Al junctions. Combined with the large sample to sample variations, we are unable to make a definitive statement regarding the CISS spin valve conductance and SOC in Cu, in contrast to Au and Al.

1. Control samples without molecules

The measurement and results of control junctions without molecules were previously presented and discussed in our prior work [1]. Since then, we have measured many more control samples with different NM electrodes (Au, Al, Ag and Cu) and are in position to place definitive upper bound on the MC in such junctions. The control samples were fabricated with the same device fabrication process omitting the assembly of molecules. The figure below shows a set of representative results, and we summarize the main points in the following.

1. *Repeated measurements on the same junction*: Similar to the molecular junctions, the “spin-valve conductance” (ΔG) of the control junctions were determined from both I-V and direct MR measurements. Supplementary Fig. 4(a) shows two sets of I-V measurements in opposite saturation fields of ±700 Oe. It is evident that the differences between the I-V’s in opposite fields and repeated measurements are minimal, essentially at noise level. This is evidenced in the extracted ΔG shown in Supplementary Fig. 4(b). The repeat measurements also evidenced a lack of well-defined bias dependence for the ΔG. Furthermore, the values of ΔG determined from direct MR measurements were not consistent with those extracted from I-V measurements; in most cases, there were not well-defined MC curves (details in SI of our previous paper  [1]). These are in stark contrast with molecular junctions with Au electrode, where the I-V’s, extracted ΔG, and their bias dependences are highly consistent, reproducible, and well-defined.
2. *Different junctions in different samples*: The minimal values and lack of consistent bias dependence of ΔG in the control junctions without molecules were also evidenced in comparison of different junctions in different control samples. Supplementary Fig. 4(c) shows ΔG of two control junctions with Au contacts. The values of ΔG are minimal without consistent bias dependence.
3. *Different NM electrodes*: Supplementary Fig. 4(d) shows a comparison of ΔG of control junctions with Au, Al, Cu and Ag electrodes. Again, in all cases, the values of ΔG are minimal without consistent bias dependence.


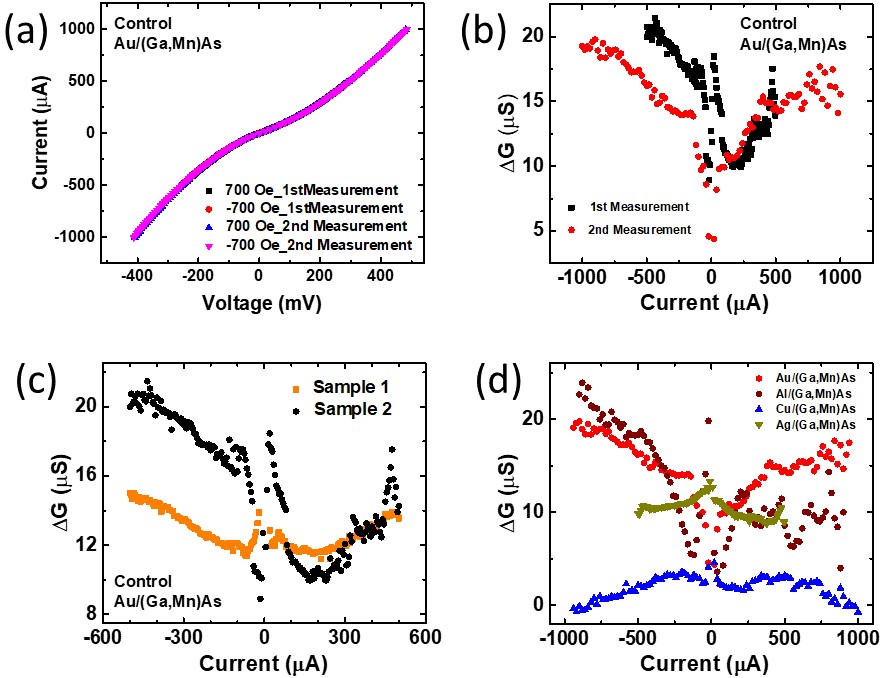


**Supplementary Fig. 4: Control samples. (a)** Two separate measurements of I-V curves for a control junction (Au/(Ga,Mn)As) without any molecules in perpendicular magnetic fields of ±700 Oe. **(b)** Bias dependence of ΔG extracted from the I-V curves in **(a). (c)** Comparison of bias-current dependent ΔG for two different control junctions with Au contacts from different samples. **(d)** Comparison of bias-current dependent ΔG of the control junctions with Au, Al, Cu and Ag electrodes.


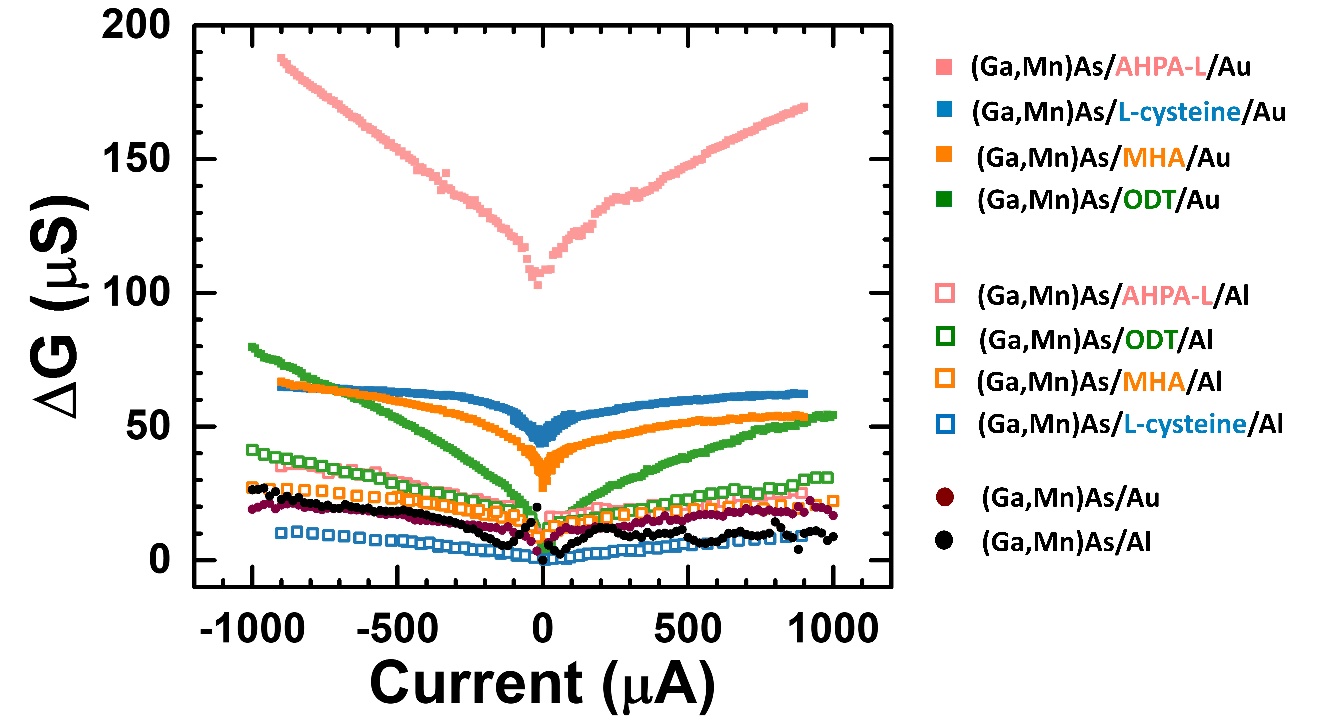


**Supplementary Fig. 5:** Comparison of the bias-dependent spin valve conductance (ΔG) of molecular junctions (chiral and achiral) with Au and Al normal metal electrodes, and that of control junctions without any molecules with Au and Al electrodes.

Taken together, the extensive measurements of MC in the control established a solid reference for meaningful comparison of the MC in various molecular junctions. Supplementary Fig. 5 shows the comparison of the bias dependences of the spin valve MC of molecular junctions (chiral and achiral) with Au and Al normal metal contacts, and those of control samples with Au and Al contacts on (Ga,Mn)As without any molecules. It is worth noting that the bias-dependent MC of the molecular junction with Al electrode is similar to that in the control junctions with both Au and Al contact, suggesting that the MC of molecular junctions with Al electrode is essentially negligible.

1. Potential contribution of AHE of (Ga,Mn)As in observed MC

Potential contribution of the anomalous Hall effect (AHE) of the (Ga,Mn)As layer to the measured junction MC was a critical first issue in our previous work [1], where we devoted significant initial efforts on detailed characterization of the (Ga,Mn)As layer and ruling out spurious effects from its magnetoresistive responses. The results were discussed in the main text of our previous paper  [1] and elaborated in its SI. Here we reiterate several key points and add a couple more based on results from current work. These results together make a compelling case against any significant contribution of AHE in the (Ga,Mn)As to the MC of molecular junctions with Au contacts.

1. The junctions in our experiments were designed and fabricated as true vertical junctions by lithography to minimize “current crowding”. While such junctions are not four-terminal devices in the strictest sense, they are very close. Several observations provide strong evidence that the contribution to the junction resistance from the (Ga,Mn)As layer is very small: 1) The (Ga,Mn)As in our devices have extremely high doping levels of several ***percent***. This results in carrier (hole) densities on the order of 10^21^ cm^-3^ and resistivities on the order of mΩ∙cm, close to those in metals. 2) The junction resistances of high-quality molecular junctions always show weakly insulating behavior, which is different from, even opposite of the temperature dependences of the longitudinal or Hall resistances of the (Ga,Mn)As film  [1].
2. The MC of the molecular junctions show nontrivial dependences on the biases. Specifically, the MC of chiral molecular junctions show approximate linear dependence on the bias current over large ranges of biases. This is qualitatively different from the bias (in)dependence of the AHE of the (Ga,Mn)As: The Hall voltage is linear with the current, and the Hall resistance or conductance is constant, i.e., independent of the current.
3. If the AHE of (Ga,Mn)As actually contributed significantly to the junction MR, we would expect the effect to be more pronounced in junctions with lower resistances, especially control junctions without molecules. In our experiments, the junction MC (ΔG) show no correlation with the total junction conductance, as detailed in Section 2 above. Also, as elaborated in Section 4 above, all control junctions showed minimal MC without well-defined bias dependence.
4. If the junction MC was dominated by spurious contribution from the AHE in the (Ga,Mn)As layer, its sign would depend on the relative orientation of the bias current and applied magnetic field. In our experiments, *the sign of the molecular junction MR depends only on the field direction regardless of the measurement configuration*, i.e., different configurations of the quasi four-terminal setup or specific current path. (We thank an anonymous reviewer whose comment led to our identification of this important point).
5. Further details on the fitting procedure

The fitting of the bias dependence of $\Delta G$ using Eq. (3) in the main text was done both in Origin and by using the curve fitting toolbox (cftool) of MATLAB. The fitting was performed separately for positive and negative bias currents, using $\alpha_{o}$, $\alpha_{-M}$ and $\alpha_{+M}$ as fitting parameters. The value of *β* was kept constant at 10 V^-1^ while fitting bias dependence of both chiral and achiral molecules. The typical process of fitting in Origin along with the parameter values, standard errors, and Adj. R-Square are shown in Supplementary Fig. 6. We point out that the resulting values of the parameters are similar for fittings done by Origin and MATLAB.


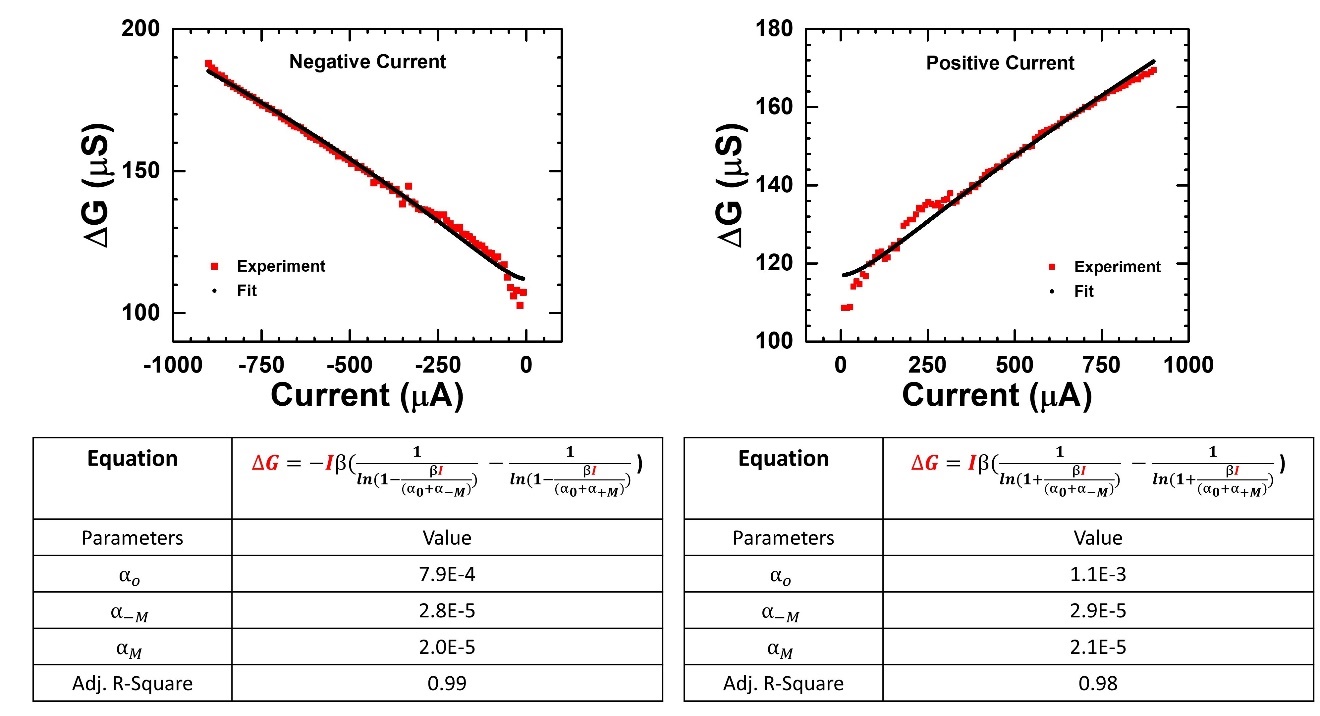


**Supplementary Fig. 6:** An example depicting the process of curve fitting of the bias-dependent ΔG in Origin. The data is from the spin-valve conductance for a (Ga,Mn)As/AHPA-L/Au junction. Similar process of fitting was employed for all molecular junctions with Au and Al contact.

1. Fitting results for junctions of achiral molecules

Using the procedure described in the previous section, fittings to Eq. (3) were performed for the achiral molecular junctions (MHA and ODT) with Au and Al normal metal electrodes. Supplementary Fig. 7 shows the best fits. Supplementary Table 1 lists the resulting values for the parameters from the best fits.


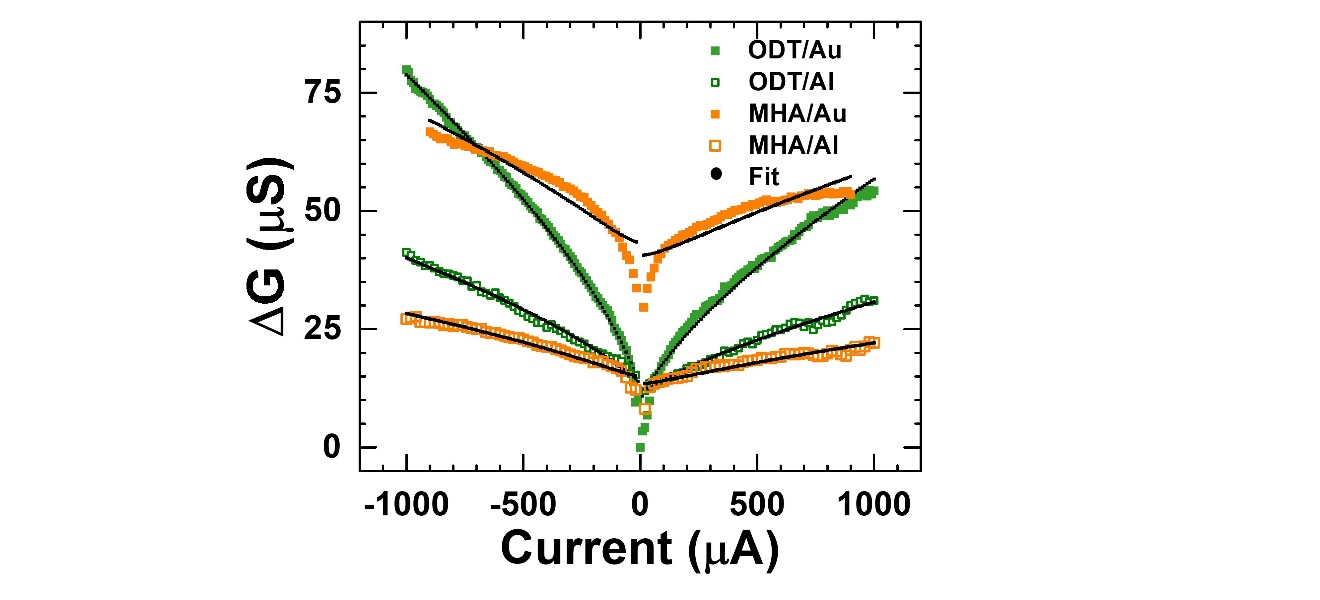


**Supplementary Fig. 7:** Fitting results of the spin valve conductance for (Ga,Mn)As/achiral (MHA and ODT)/NM junctions with Au and Al contact.

| Achiral molecules (MHA and ODT) | | | | | | |
| --- | --- | --- | --- | --- | --- | --- |
| Junction | Negative current | | | Positive current | | |
|  | $\alpha_{o}$ | $\alpha_{-M}$ | $\alpha_{+M}$ | $\alpha_{o}$ | $\alpha_{-M}$ | $\alpha_{+M}$ |
| ODT/Au | 98.6 µS | 8.63 µS | -6.72 µS | 120 µS | 7.52 µS | -6.83 µS |
| ODT/Al | 312 µS | 3.80 µS | -2.74 µS | 421 µS | 2.31 µS | -3.53 µS |
| MHA/Au | 676 µS | 8.84 µS | -9.97 µS | 831 µS | 8.16 µS | -9.47 µS |
| MHA/Al | 254 µS | 1.89 µS | -3.29 µS | 305 µS | 2.13 µS | -3.07 µS |

Supplementary Table 1: Fitting parameters of the CISS spin valve conductance for the achiral molecular junctions with Au and Al contacts.

The values of the parameters for spin valve conductance of achiral molecular junctions obtained from the fitting are consistent with our expectation. The notable aspect of this fitting is that the values of $\alpha_{\mp M}$ in the junction with Au are much greater than those with Al, even though the values are in the same order. This is consistent with the observation of diminished but finite spin valve conductance in achiral molecules with Au contact. The values of $\alpha_{o}$ are similar to that of chiral molecular junctions.

1. Bias-induced changes in MC and I-V

Figures S8(a) and (b) show the I-V’s and bias current-dependent MC, respectively, of a (Ga,Mn)As/AHPA-L/Au junction, after the first-time application of maximum bias current of 800 μA and 1 mA. The MC results were presented previously (Supporting Figure 5, Ref. [1]). Evidently, I-V, 𝐺_𝐽_, and Δ𝐺_𝐽_ changed abruptly when the maximum applied bias current was increased from 800 μA to 1 mA for the first time. The results suggest bias-induced changes to I-V and MC when the bias voltage or current exceeds a critical value.


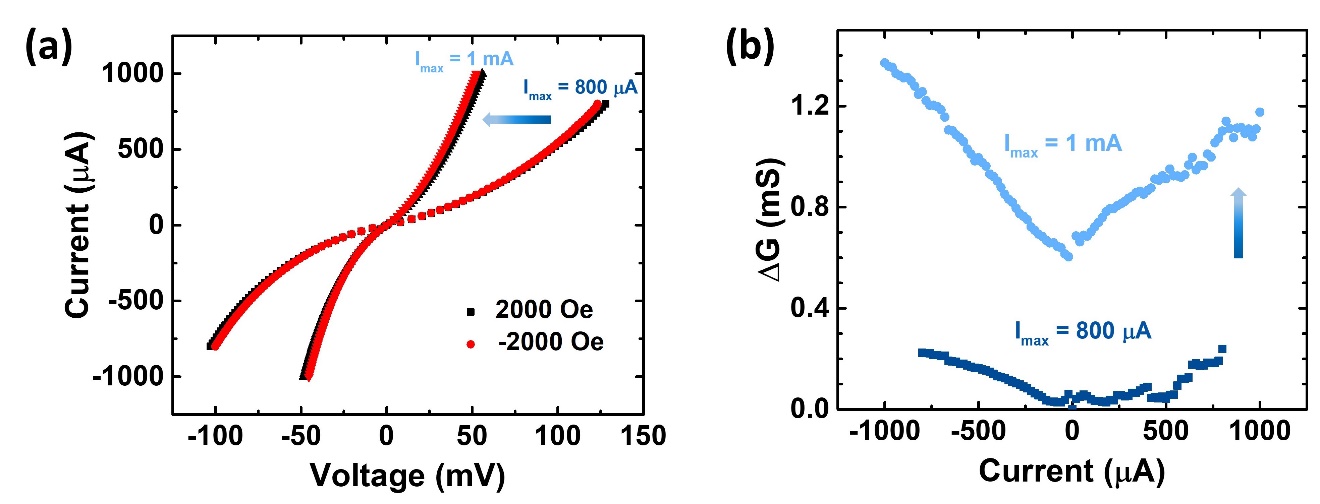


**Supplementary Fig. 8:** The I-V **(a)** and bias current-dependent MC **(b)** of a (Ga,Mn)As/AHPA-L/Au junction of size 5×5 μm^2^. The I-V changed and Δ𝐺_𝐽_ increased abruptly when the first-time maximum applied bias current was increased from 800 μA to 1 mA. All measurements were performed at 4.3 K.

**References:**

[1] T. Liu, X. Wang, H. Wang, G. Shi, F. Gao, H. Feng, H. Deng, L. Hu, E. Lochner, P. Schlottmann, S. von Molnár, J. Zhao, and P. Xiong, “Linear and Nonlinear Two-Terminal Spin-Valve Effect from Chirality-Induced Spin Selectivity”, *ACS Nano* 14, 15983 (2020).
